# Facile synthesis of highly porous CuO nanoplates (NPs) for ultrasensitive and highly selective nitrogen dioxide/nitrite sensing†

**DOI:** 10.1039/c8ra09299k

**Published:** 2019-02-15

**Authors:** Shivsharan M. Mali, Shankar S. Narwade, Yuraj H. Navale, Vikas. B. Patil, Bhaskar R. Sathe

**Affiliations:** Department of Chemistry, Dr Babasaheb Ambedkar Marathwada University Aurangabad 431004 India bhaskarsathe@gmail.com; Functional Materials Research Laboratory, School of Physical Sciences, Solapur University Solapur 413255 India

## Abstract

Copper oxide (CuO) nanoplates (NPs of ∼100 nm width) were successfully synthesized *via* a chemical method (emulsion method). Superior catalytic activities towards both chemical and electrochemical sensing of nitrite were achieved.

## Abstract 2

Copper oxide (CuO) nanoplates (NPs of ∼100 nm width) have been successfully synthesized by using a chemical method (emulsion method). Superior catalytic activities towards both chemical and electrochemical sensing of nitrite were achieved.

## Graphical abstract

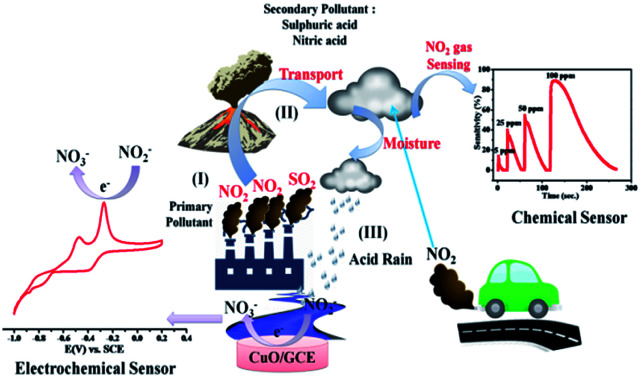

In recent years, the living standards of humans have grown immensely due to the industrial revolution. However, excessive industrialization has also caused a negative impact on human health due to the environmental degradation caused by the release of toxic gases. Air pollution is becoming more and more serious due to the increase in the concentration of toxic gases like sulphur dioxide (SO_2_), carbon monoxide (CO), ammonia (NH_3_), carbon dioxide (CO_2_), nitrogen dioxide (NO_2_), and hydrocarbons (CH)_*x*_,^1,2^ which directly impact human health. Among these toxic gases, NO_2_ is a common air pollutant that causes respiratory diseases such as emphysema and bronchitis and can aggravate existing heart diseases. (NO)_*x*_ is a family of poisonous and highly reactive gases emitted by various non-road vehicles, automobiles, trucks (*e.g.*, boats, construction, and other equipments) as well as industrial sources such as power plants, industrial boilers, cement kilns, turbines and fertilizer industries. NO_*x*_ is a strong oxidizing agent and plays a major role in the characteristic reactions with volatile organic compounds (VOC). In addition, NO_2_ gas is a potential source for nitrous and nitric acid that are responsible for acid rains, which result in the destruction of the ozone layer in the troposphere.^3^ Consequently, to detect the highly toxic NO_2_ gas, there is a need to develop low cost, highly sensitive, reliable and reproducible gas sensor systems.

Many transition metal oxides (WO_3_, TiO_2_, CuO, ZnO, MoO_3_ and many more) have been broadly used in the field of environmental monitoring, military technologies, safety engineering, and others.^4–8^ Among the various transition metal oxides, CuO has been widely studied because of its size-tunable surface features that can be utilized as gas sensors having long term stability with selectivity at comparatively low temperatures. Moreover, CuO being a wide band gap p-type semiconductor metal oxide exhibits excellent sensitivity towards gas sensing. The typical p-type semiconducting metal oxides, such as nickel oxide (NiO), possess distinct characteristics.^9^ Nitrite ions (NO_2_^−^) are hazardous, which widely exist in nature, food, physiological and manufacturing systems.^10^ Under the weakly acidic conditions in the stomach, nitrite is simply transformed into carcinogenic *N*-nitrosamines when it combines with tertiary amines present in food; this becomes the most vital reason for causing gastric cancer.^11^ It induces disintegration when dissolved in water and can act as an environmentally harmful genus for the degradation of some important fertilizers in soil. Moreover, nitrite is often used as an additive in food products because it can protect against harmful microorganisms that cause food poisoning. Therefore, it is essential to detect and examine the allowed concentration levels of nitrite ions in both physiological and environmental systems.^12^ In current years, many precise analytical techniques, such as gas chromatography,^13^ spectrophotometry,^14^ chemiluminescence,^15^ capillary electrophoresis,^16^ high performance liquid chromatography^17^ and electrochemical methods,^18^ have been developed and utilized to investigate the nitrite ion concentration, which is crucial for environmental and human health.

Among the various noble metal and metal oxide NPs, CuO NPs have gained widespread attention for various applications because of their low cost, high catalytic, optical, antimicrobial and electrical conductivity properties.^19^ Highly stable CuO NPs were synthesized in a facile manner through self-oxidation of the surface using a chemical emulsion method and subsequent calcination at 400 °C (Scheme 1). The resulting NPs were utilized further for environmental monitoring of NO_2_ by chemical and electrochemical approaches. The detailed synthesis procedure and other supporting experimental details are presented in ESI (ESI-I†) and are shown schematically in Scheme S1.†

**Scheme 1:**
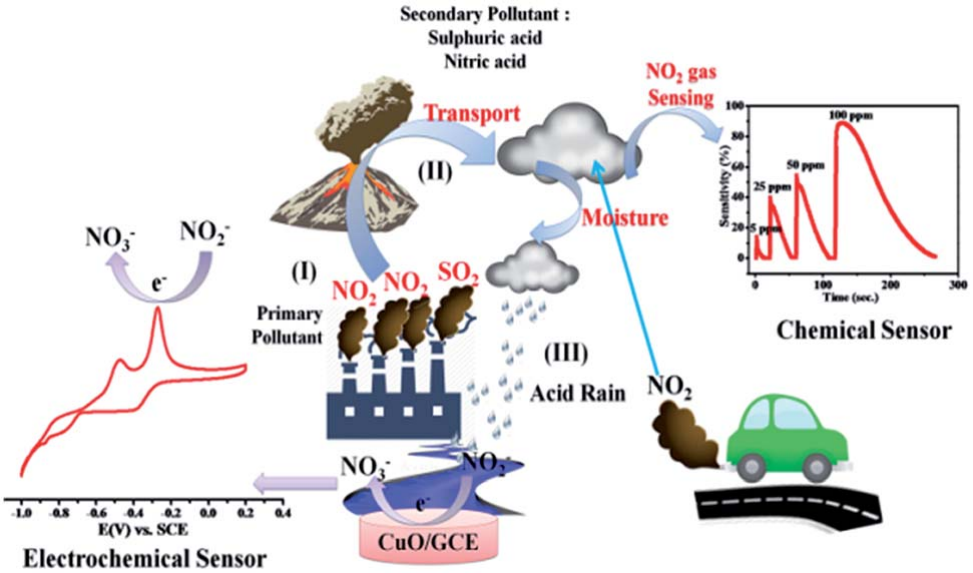
Environmental monitoring of NO_2_ using both chemical and electrochemical methods.

Accordingly, the surface morphology of the chemically synthesised CuO NPs was examined by FESEM. As can be seen in Fig. 1(a) and (b), a large yield of homogenously dispersed NPs was obtained. These NPs are interconnected with each other and are consistently distributed with clear edges. The obtained dimensions of NPs varied between edge widths of ∼100 nm to ∼500 nm, as confirmed by the TEM analysis shown in the ESI.† This type of morphology is quite different from the morphology reported for other NPs in the literature,^20^ which could be due to the role of PVP as a surface directing molecule and the reaction conditions maintained during nucleation followed by growth. The XRD pattern shown in Fig. 1(c) illustrates the characteristic peaks of CuO indicating its crystalline nature. Considerably, the peaks at 2*θ* values match with the crystal planes of (110), (−111), (111), (−202), (020), (202), (−113), (022), (220), (113) and (203), and are in good agreement with the previous report on a similar system.^21^ Furthermore, the diffraction peaks have also been confirmed with the JCPDS card # 00-045-0937, indicating that the structure of the CuO NPs is hexagonal. No representative characteristic peaks for other impurity phases were detected, suggesting that the high quality single phase of CuO NPs was formed. The average crystallite size (*d* = ∼100 nm) of CuO NPs was estimated by Scherrer's formula and is in good agreement with the morphological findings from SEM.

**Fig. 1:**
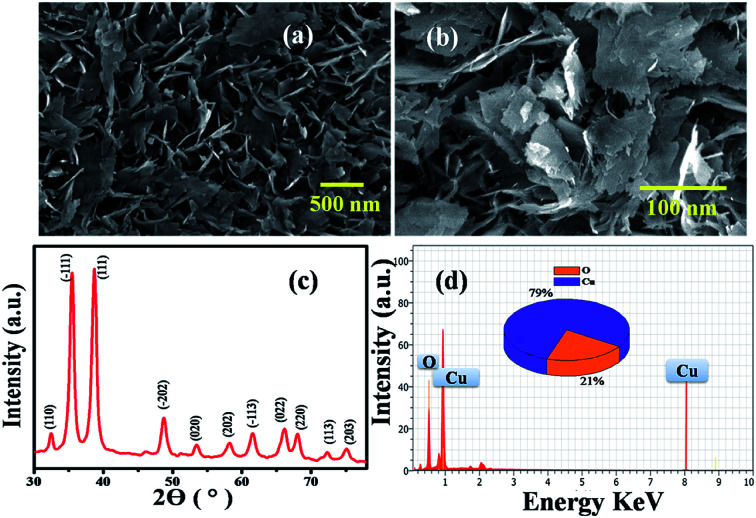
(a) and (b) SEM images of CuO NPs having an edge width of ∼100 nm. (c) X-ray diffraction pattern of (110), (−111), (111), (−202), (020), (202), (−113), (022), (220), (113), (203) corresponding to CuO NPs having a hexagonal crystal structure (d) EDAX of CuO nanoplates.

Further, the EDAX analysis (Fig. 1(d)) shows the signals at 1.01 keV and 8.06 keV corresponding to Cu and the signal at 0.29 keV corresponding to O, clearly indicating the 79 : 21 ratio of Cu : O, which is in good agreement with the theoretical ratio for CuO reported in literature.^22^ These NPs were further characterized by TGA (Fig. S1†) and BET (Fig. S2†) surface area measurements. Accordingly, the thermogram depicts the weight loss (*w*_1_) of 0.5 to 1% below 150 °C corresponding to physically absorbed water. The weight drop (*w*_2_) of almost (1%) from 200–500 °C corresponds to the decomposition of Cu(ii) to CuO. Further weight loss (*w*_3_) above 500 °C can be attributed to the thermal degradation of the surfactant, leaving 2.5–10% of CuO in the N_2_ atmosphere. Moreover, the BET profile of CuO NPs (Fig. S2†) displays a type II isotherm with a hysteresis loop in the relative pressure (*P*/*P*_0_) range from 0.8 to 0.9, indicating its mesoporous nature that could be due to the thermal decomposition of polyvinyl pyrrolidone (PVP) on the CuO surface. The BET (Brunauer–Emmett–Teller) surface area calculated from the adsorption branch of the isotherm is found to be 23.60 m^2^ g^−1^. The inset in Fig. S2† illustrates the corresponding pore size distribution plot calculated by BJH (Barrett–Joyner–Halenda) from the adsorption data. The average pore radius and pore volume of the CuO NPS was calculated to be 2.3 nm and 2.599 × 10^−0.1^ cm^3^ g^−1^, showing that the particles have a high surface area from its nanodimensions and features.

The plot of decreasing electrical resistance of the CuO NPs sensor upon the exposure of 100 ppm oxidizing NO_2_ gas at 150 °C is shown in Fig. 2(a). The recovery of the sensor was recorded by exposing the sensor to air. From the response curves, the response% (*S*) was calculated using the following relation:1
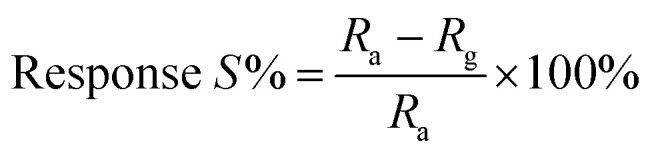
where, *R*_g_ and *R*_a_ are the resistances of the film in the presence of NO_2_ and absence of NO_2_ (in air), respectively. The response and recovery times were defined as the times needed for a 90% total resistance change on exposure to gas and air, respectively.^23^ The response time is the time over which the resistance reaches a fixed percentage (usually 90%) of the final value when the sensor is exposed to the full scale gas concentration. The time response is particularly dependent on the sensor properties, such as electrode geometry, crystallite size, additives, diffusion rates, and electrode position. A shorter response time is indicative of a good sensor. The recovery time is the time interval over which the sensor resistance reduces to 10% of the saturation value when the sensor is exposed to the full scale concentration of the gas and then exposed to clean air. A good sensor should have a small recovery time so that the sensor can be used repeatedly.^23^ It has been observed that the operating temperature plays an important role in the gas sensing performance, which influences the adsorption/desorption process of oxygen ions on the CuO surface. The metal oxide-based sensors adsorb oxygen from air and form O^−^, O_2_^−^ and O^2−^ species. The experimental results from the sensing response with respect to the operating temperature in the range of 150–250 °C is shown in Fig. 2(b). The plot exhibits a maximum response value of 88% at the operating temperature of 150 °C towards 100 ppm NO_2_ gas. The response of the CuO NPs sensor is restricted at lower temperatures (below 150 °C), which could be due to the low rate of diffusion of NO_2_ gas at the sensor surface; however, at higher temperatures (above 150 °C) the rate of diffusion of NO_2_ gas molecules increases. Thus, for the further gas sensing studies, 150 °C is used as the optimized sensing temperature for CuO NPs sensors.

**Fig. 2:**
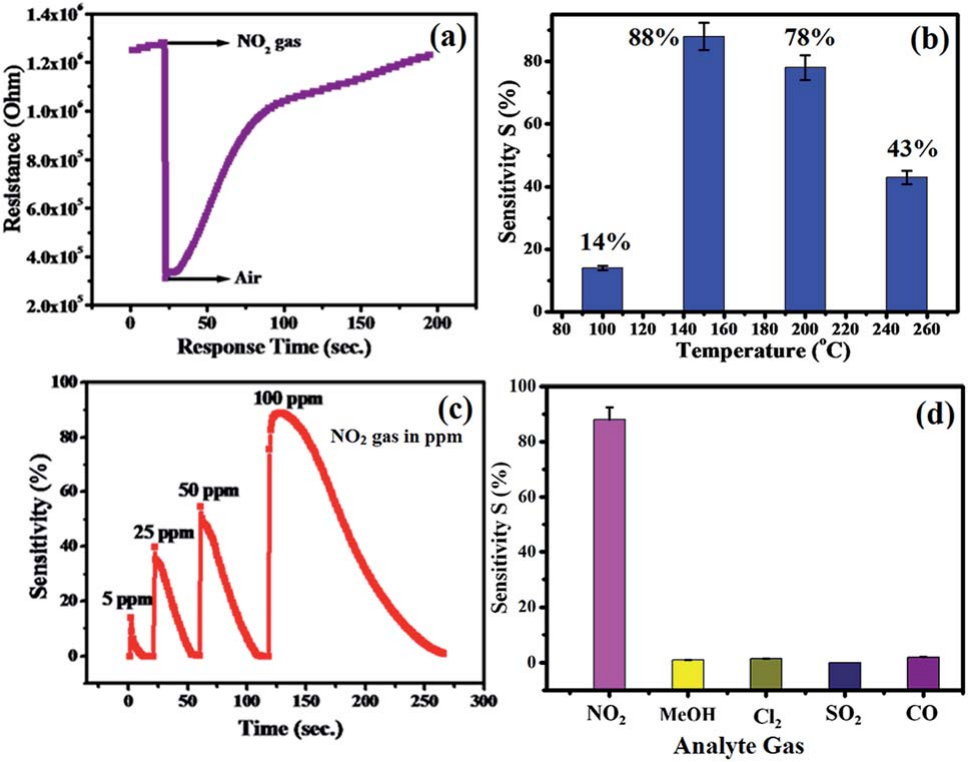
(a) Variation in resistance of CuO NPs-based films with respect to time in contact with NO_2_ gas (b) temperature dependent response of CuO for NO_2_ gas sensing (c) energetic response of CuO NPs sensor towards 5, 25, 50 and 100 ppm NO_2_ gas concentrations at the same time and (d) gas selectivity of the CuO NPs sensor to other gas species.

The response curve of the CuO NPs sensor with respect to different concentrations (5–100 ppm) of NO_2_ gas at 150 °C is shown in Fig. 2(c) and demonstrates that the response values of the sensor increase with an increase in the concentration of NO_2_ gas. For example, at 5 ppm of NO_2_, the response of the sensor was observed as ∼14%, whereas a maximum response value of 88% toward 100 ppm NO_2_ at 150 °C was recorded. This could be due to decreased contact between the sensor surface of CuO NPs and NO_2_ gas at lower concentrations, which subsequently led to a smaller response value. Conversely, at high concentrations of NO_2_ gas, the gas molecules cover more of the CuO NPs surface. As a result, a higher response value was achieved due to the greater surface interactions.^24^ Furthermore, as shown in Fig. 2(d), there were minimal changes in the selectivity of the CuO NPs towards different target gases at a fixed 100 ppm concentration of each of them. Selectivity studies evidently suggest that the CuO NPs are more sensitive towards NO_2_ gas compared to other test gases, *viz.*, liquefied CO, SO_2_, CH_3_OH, and Cl_2_. This high selectivity towards NO_2_ is probably due to the greater rate of reaction between the CuO NPs surface and NO_2_ gas molecules compared to those of the other gases.

Furthermore, a comparison of gas-sensing parameters of the present compound with those reported in the literature for NO_2_ gas is shown in Table 1.

**Table 1:** Comparison of performance of the as-synthesized CuO NPs with some representative metal oxide-based NO_2_ gas sensors from the literature

Materials	Synthesis method	Operating temperature (°C)	Gas response %	Ref. no.
CuO nanowire	Thermal oxidation	200	8.9 for 5 ppm	25
CuO nanoparticle	Thermal evaporation	150	5 for 5 ppm	26
CuO nanoparticle	Chemical bath deposition	200	3.5 for 5 ppm	27
CuO nanoparticle	Solid state reaction method	600	18 for 25 ppm	28
Ag–CuO NPs	Solid state reaction method	650	5 for 25 ppm	29
CuO nanofiber	Electrospinning method	400	4 for 10 ppm	30
MoO_3_–V_2_O_5_	Chemical spray pyrolysis	200	25 for 20 ppm	31
Aluminium doped ZnO thin films	Sol–gel method	200	5 for 5 ppm	32
Nb_2_O_5_-SE	Sol–gel method	780	17 for 50 ppm	33
ZnO nanorods	Hydrothermal	200	5 for 10 ppm	34
(Ni, Co, Fe)–SnO_2_	Chemical method	650	4–14 for 10 ppm	35
CuO nanoplates	Chemical method	150	14 for 5 ppm	Present work

In general, the semiconducting metal oxide-based gas sensing mechanism is predominantly associated with the change in electrical resistance due to contact of the target gases.^36^ The variation in electrical resistance of the sensor is principally due to the adsorption/desorption processes taking place with target gases on the surface. CuO is a p-type semiconductor that contains holes as the bulk charge carriers. When the CuO NPs sensor is exposed to normal atmospheric air, the oxygen molecules from the air adsorb on the peak of the sensor surface, which leads to an increase in the adsorbed oxygen species (Scheme 1).

2 CuO + O_2_ → (CuO)·2O^−^

The reaction between the adsorbed NO_2_ gas molecules and CuO NPs is presented schematically in Fig. 3(a). NO_2_ is an oxidizing gas (electron tolerant in nature) and its contact with the p-type CuO leads to the rise in conductivity due to the enhancement in hole concentration in the conduction band of p-type CuO by decreasing the rate of resistance. Upon exposure to NO_2_ gas, the probable reaction occurring at the CuO NPs surface is given below:

3 (CuO)·2O + NO_2_ → (CuO)·O^2−^_2_ + NO↑

**Fig. 3:**
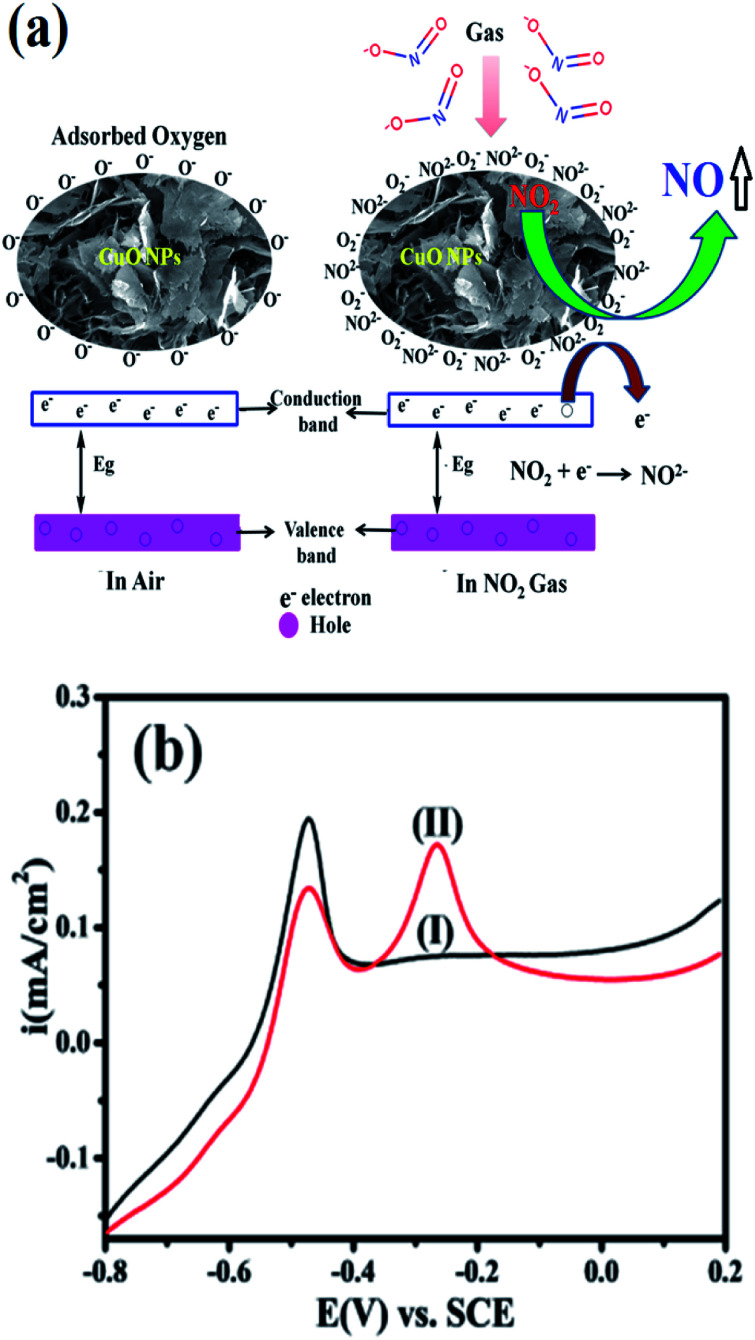
(a) Scheme showing the mechanism of the CuO NPs sensor for NO_2_ gas sensing, where an electron transfers to the air and a NO_2_ gas molecule hole–electron pair transfers from the VB to CB. (b) Superimposed anodic segments for GC modified by CuO in (I) 0.5 M KOH (black), and (II) of the same with 20 μM nitrite in 0.5 M KOH (red).

It is known that the NO_2_ sensing mechanism on CuO NPs depends on the active surface oxygen centres that exist on the CuO NPs surface. Linear sweep voltammetric (LSV) experiments on the electrochemical oxidation of nitrite were carried out to further determine/support the earlier findings of oxidative sensing on the CuO surface. Accordingly, the limit of detection (LOD) of our systems with results from literature compared in Table 2.

**Table 2:** Correlation of analytical performance of different electrochemical sensors for nitrite (NO_2_^−^) detection

Sensor material	Analytical technique	Limit of detection (LOD) μM	Ref.
PPy-NWs	CV	50	38
f-ZnO@rFGO	LSV	33	39
ZnTiO_3_–TiO_2_	Amperometry	3.98	40
Graphene-nafion/GCE	CV	11.61	41
EPPGE-MWCNT-PB	Amperometry	6.1	42
rGO-Co_3_O_4_@Pt/GCE	CV	1.73	43
**CuO NPs**	****LS**V**	**1.1**	**Present work**

The selectivity study of a CuO NPs-based gas sensor was performed for nitrogen dioxide (NO_2_), methanol (CH_3_OH), chlorine (Cl_2_), carbon monoxide (CO) and sulphur dioxide (SO_2_) at 100 ppm concentrations of each gas, as shown in Fig. 2(d). The resistance of the CuO film sensor did not change for Cl_2_, CO, SO_2_ and CH_3_OH gases, hence, the response was considered to be minor, confirming that these gases were not interacting with the CuO sensor. Consequently, the metal oxide-based gas sensor works on the principle of chemiresistance, *viz.*, the change in resistivity or electrical conductivity of thin films upon contact with the target gas. The gas molecules interacting with the metal oxides either act as a donor or an acceptor of charge carriers (receptor function), and alter the resistivity of the metal oxide. The decrease or increase in the resistance of the metal oxide thin film depends upon the type of majority carriers in the oxide film and also the nature of the gas molecules (whether oxidizing or reducing) in an ambient atmosphere.^9^ For n-type materials, oxidizing gases (acceptors) increase the resistance of the thin film, while reducing gases (donors) decrease the resistance and are correspondingly converse for p-type materials. The binding energies also confirm that the physisorption occurs between the molecule and the surface for CH_3_OH, CO, SO_2_, and Cl_2_, with NO_2_ being the most strongly bound species. The binding energy for NO_2_ is approximately greater than those of the other gas molecules. On exposure to NO_2_ oxidizing gas, the CuO film resistance decreases, suggesting a p-type conduction behaviour of CuO. The NO_2_ sensing of CuO depends on the surface oxygen adsorbed on the CuO NPs surface. The sensing device involves the adsorption of oxygen species on the surface of CuO NPs, which results in more electron density, and thus, causes a decrease in the potential barrier at the grain boundaries. The gas molecules interact with the oxygen species and produce a notable change in the electronic properties of the material. Thus, the density of oxygen species on the surface defines the rate of reaction and the catalytic properties. NO_2_ is an oxidizing gas with an electron affinity much higher than that of oxygen (0.48 eV); NO_2_ can interact with CuO by trapping electrons directly through the surface oxygen ions thereby forming new surface electron acceptor levels.^8^

Accordingly, Fig. 3(b) shows a comparison of the CuO in 20 μM nitrite in 0.5 M KOH at a scan rate of 50 mV s^−1^. It is clearly seen that the modified CuO/GC (black line) in 0.5 M KOH displayed no signal corresponding to nitrate. However, in the presence of 20 μM nitrate (red line) in 0.5 M KOH, a fine additional oxidation peak at −0.30 V *vs.* SCE corresponding to nitrate is observed. This sensitivity can be attributed to the large surface area created by stabilized CuO NPs, which makes it easier for the adsorption of nitrite and provides adequate and successful reaction sites. These results extensively reveal how the electrocatalytic activity of CuO NPs in an aqueous system may afford an indirect link *via* NO_2_^−^ oxidation and confirms the electrochemical sensing capability of CuO NPs towards NO_2_^−^ determination as one of the intermediate species of NO_2_ in aqueous systems. Furthermore, the influence of the increase in concentration of NO_2_^−^ on the electrocatalytic oxidation on CuO NPs in 0.5 M KOH was studied using an LSV segment in Fig. S4(a).†

The oxidation peak current at −0.30 V *vs.* RCE shows a linear response with the increase in concentration of NO_2_^−^ ions in the range of 10–50 μM and this linear range is much broader than those of the reported comparable electrocatalytic systems. Furthermore, the influence of the scan rate on the electrocatalytic oxidation peak potential (Epa) and peak current for a 20 μM concentration of NO_2_^−^ at the CuO/GCE electrode in 0.5 M KOH was studied using LSV, as shown in Fig. S4(b).† The current values were observed to increase with an increase in the scan rate from 10 to 100 mV s^−1^. The linear association between the anodic peak currents and the square root of the scan rate^37^ showed that the electro-oxidation of NO_2_^−^ is diffusion-controlled.

The overall LSV result for the electrochemical mechanism^37^ of nitrite ions (NO_2_^−^) involves a reversible charge transfer reaction (eqn (4)), the obtained NO_2_ disproportionation into NO_3_^−^ and NO_2_^−^ (eqn (5)), and in the end, the NO_2_^−^ undergoing a unidirectional reaction for the oxidation of nitrite at the CuO modified GC electrode in an irreversible approach (eqn (6)).^44–48^ This is in good agreement with the above presented chemical sensing studies on CuO in the solid state.

4 2NO^−^_2_ ⇋ NO_2_ + 2e^−^

5 2NO_2_ + H_2_O → NO^−^_2_ + NO^−^_3_

6 NO^−^_2_ + H_2_O → NO^−^_3_ + 2H^+^ + 2e^−^

In conclusion, CuO NPs were synthesized using a chemical method (emulsion) and their gas sensing activity was studied towards a series of gases. The gas sensing studies revealed that the CuO NPs sensor was capable of detecting very low concentrations (5 ppm) of NO_2_ gas at a low operating temperature of 150 °C. The CuO NPs sensor exhibited a maximum response value of 88% upon exposure to 100 ppm NO_2_ gas. The change in electrical resistance of the CuO NPs sensor following the interaction of NO_2_ gas molecules was mainly contributed by the adsorbed oxygen species at the grain boundaries. Moreover the fabricated CuO/GC modified electrode was effectively applied for electrochemical nitrite sensing. It was found that Cu NPs showed improved electrocatalytic behaviour towards nitrite. This proposed sensor exhibited a wide linear range with high sensitivity and low detection limit along with fast response/recovery time, excellent repeatability and stability.

## Conflicts of interest

There are no conflicts to declare.

## Supplementary Material

RA-009-C8RA09299K-s001
