# Supplementary material for: Facile synthesis of highly porous CuO nanoplates (NPs) for ultrasensitive and highly selective nitrogen dioxide/nitrite sensing
Source: RSC Adv. 2019 Feb 15;9(10):5742–7. doi: 10.1039/c8ra09299k (PMC9060789; doi:10.1039/c8ra09299k)
Supplement: RA-009-C8RA09299K-s001 [file RA-009-C8RA09299K-s001.pdf]

Electronic Supplementary Material (ESI) for RSC Advances.
This journal is © The Royal Society of Chemistry 2019

Supplementary Information

Facile Synthesis of Highly Porous CuO Nanoplates (NPs) for Ultrasensitive and Highly Selective Nitrogen Dioxide/Nitrite Sensing

Shivsharan M. Mali[a], Shankar S. Narwade[a], Yuraj H. Navale[b], Vikas. B. Patil[b], Bhaskar R. Sathe[a*]

Department of Chemistry, Dr. Babasaheb Ambedkar Marathwada University, Aurangabad, 431004, India

Corresponding author E-mail: bhaskarsathe@gmail.com

Functional Materials Research Laboratory, School of Physical Sciences, Solapur University, Solapur, 413255, India

SI-I: Synthesis Procedure and Experimental Details:

All the electrochemical measurements were carried out using CHI-660E (CH instruments USA) electrochemical workstation with a three electrode system. A glassy carbon electrode (GCE) of 3 mm diameter was used as working electrode; a platinum wire and saturated calomel electrode (SCE) were used as the counter and reference electrodes respectively. All potentials are referred to SCE (3 M KCl).

In the present work the CuO NPs has been synthesised via low temperature emulsion synthesis method (chemical). Films of CuO were made-up on glass substrates using simple doctor blade method and characterized using FESEM, XRD, EDS, BET and TGA techniques. The gas sensing characteristics of CuO films were carried out towards Cl_2, NO_2, CO, SO_2, and CH_3OH gases at an optimized temperature of 150 ^oC. Further, other gas sensing parameters such as stability, reproducibility, linearity and selectivity of CuO films were

systematically studied and explored. Here we made an effort to representation the probable sensing mechanism of CuO thin films towards NO_2 gas via band structure model. Furthermore, the obtained results may give new direction for optimizing the conditions to increase the performance of CuO films in device applications. On the other hand the electrochemical applications to synthesised CuO NPs used to relevant for electrochemical detection of nitrate in aqueous medium. The use of CuO modified GC electrode for the determination of nitrite is demonstrated and subsequently applied for nitrite sensing in real samples.

Copper acetate 99.99% (Alfa-Aesar), polyvinyl pyrrolidone (PVP), polyvinyl alcohol (PVA), Sodium nitrite, Ammonia solution 25%, Sodium hydroxide and Acetone. All organic and inorganic chemicals were purchased analytical grade and used without any further purification. Deionised water (18M Ώ) from Milli-Q system was used for all synthesis and electrochemical evaluation of electrocatalytic materials.

Copper oxide (CuO) NPs is synthesized by cheap emulsion method. In a 250 mL round bottom flask 0.1M $Cu(Ac)_2$ as a source of 'Cu' and 0.01 M of PVP (Polyvinyl pyrrolidone) were added with 100 mL DI water and the reaction mixture was stirred vigorously at room temperature for 30 min leading to the formation of Cu acetate and PVP mixture. This mixture was transfer in to the oil bath and added drop wise ammonia percentage up to the pH 10-12 & refluxed to obtain brownish black precipitate. The prepared powder was washed several times with DI water and methanol to eliminate the salt and dried in air at 100 ^{o}C for 1hr. The prepared powder was then located in alumina boat and calcined at 400 ^{o}C for 1hr and consequently cooled to room temperature. For thin film formation, CuO powder was dissolved in polyvinyl alcohol (PVA) solution as a role of binder and constantly stirred for 12 hr to get solution. The obtained uniform solution of CuO was deposited on glass

substrate using Doctor Blade method and dried in air atmosphere. This film was then heated at 400 ^{o}C for 2 hr to remove PVA binder and then used for gas sensing application.

XRD patterns of CuO samples are recorded by using X-ray diffraction method equipped with strong Cu Kα1 radiation (λ = 1.54 Å), at a scanning rate of 2 degree per min in the scanning range of 2θ from 20°- 80°. The CuO NPs were drop coated over a carbon tape and further the samples were then sputter coated with Pt prior to their characterization using scanning electron microscopy (FE-SEM, JEOL, and Japan) to avoid charging effect. BET surface area analysis has been performed using AutosorbiQ (Quantachrome Inc., USA) gas sorption system. Low pressure volumetric N_2 adsorption-desorption measurements were performed at 77 K maintained by low temperature liquid N_2 bath with pressure ranging from 0-760 torr. Out gassing route was carried out at 200 °C for 15 h under dynamic vacuum (10-3torr) until a stable weight was achieved. Ultrahigh purity grade (99.999 %) N_2 was used, which is further purified by using calcium aluminosilicate adsorbents to remove the trace amounts of water and other impurities prior to the measurements. For N_2 isotherms, warm and cold free-space correction measurements were performed with ultrahigh pure He gas (99.999% purity). For the measurement, about 200 mg of samples were used and to confirm complete elimination of all guest H_2O molecules from the samples, weight of the samples was measured before and after out gassing process. Specific surface area was calculated by Brunauer-Emmett-Teller (BET) method. The TGA thermogram was obtained using TG Analyzer (SDT Q600 V20.9 Build 20) in the temperature range from 25 to 600 ^{o}C with heating rate of 10 °C/min in N_2 atmosphere.

The thin films of CuO NPs are tested for chemical gas sensing assets. The electrical connections of silver paste separated by 1cm were layered on CuO thin films. The sensor was mounted in a stainless-steel test chamber (volume: 250 cm3). A required concentration of the

test gas in the chamber was achieved by injecting a known quantity of gas using a micro syringe. A change in resistance of the film as a function of time (response curve) was recorded at operating temperature of 200 °C for 5, 25, 50, and 100 ppm concentration of NO_2 gas, which was commercially procured. The response data was acquired by using a computer interfaced Keithley 6514 system electrometer. The recovery of the sensor was recorded by exposing the sensor to air. From the response curves, the response % (S) was calculated using the following relation:

$$Response\ S\ \% = \frac{R_a - R_g}{R_a} \times 100\% \ldots\ldots\ldots\ldots 1$$

Where, Rg and Ra are resistances of film in the occurrence of NO_2 and without NO_2 (in air) respectively. Response and recovery times were defined as the time required for 90% of total resistance change on contact to gas and air, respectively.

All electrochemical measurements were carried out with a CHI-660E (CH instruments USA) instrument using a predictable three-electrode system, where a saturated calomel electrode (SCE) served as the reference electrode, a platinum wire electrode as the counter electrode and a customized GCE as the working electrode. A 3 mm diameter. Glassy carbon (GC) electrode was polished using 0.3 and 0.05 mm alumina powders, followed by washing with distilled water and methanol to remove inorganic and organic impurities. The working electrode was prepared using 10 mL aliquot of the slurry made by sonication of 1mg of the CuO NPs in 1mL isopropyl alcohol and then drop-casted on to the GC electrode. After this 2 mL of 0.01 wt% Nafion diluted with ethanol was coated on the surface of the electrocatalytic CuO NP layer to yield a uniform thin film. This electrode was then dried in air and was used as the working electrode for all electrochemical studies. An aqueous solution of 0.5 M KOH was used as the electrolyte during the electrochemical studies. Further, the nitrite NO_2- oxidation studies were performed using different concentrations of 10 to 50 μM NO_2 in 0.5

M KOH as the supporting electrolyte on CuO NP based cost effective electrocatalytic systems. LSV of CuO/GCE in 0.5 M KOH containing 20 μM NO_2 was carried out at different scan rates (10-100 mV/s).

Schematic for Synthesis:

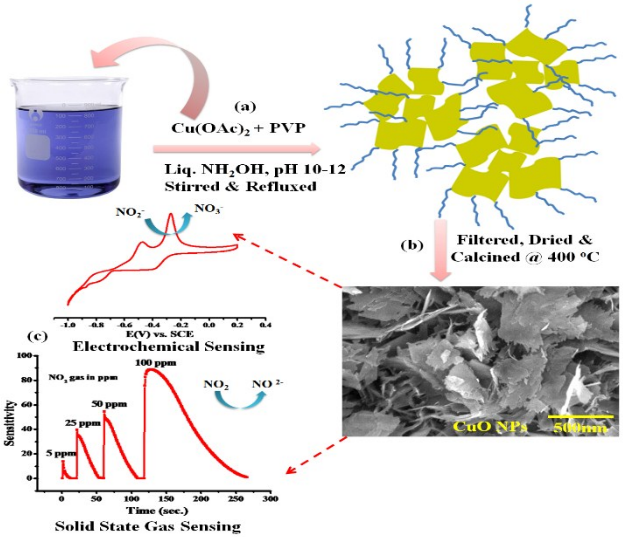

Scheme 1: Chemical synthesis of CuO NPs and there environmental monitoring NO_2 sensing studies for both chemical and electrochemical.

TEM analysis:

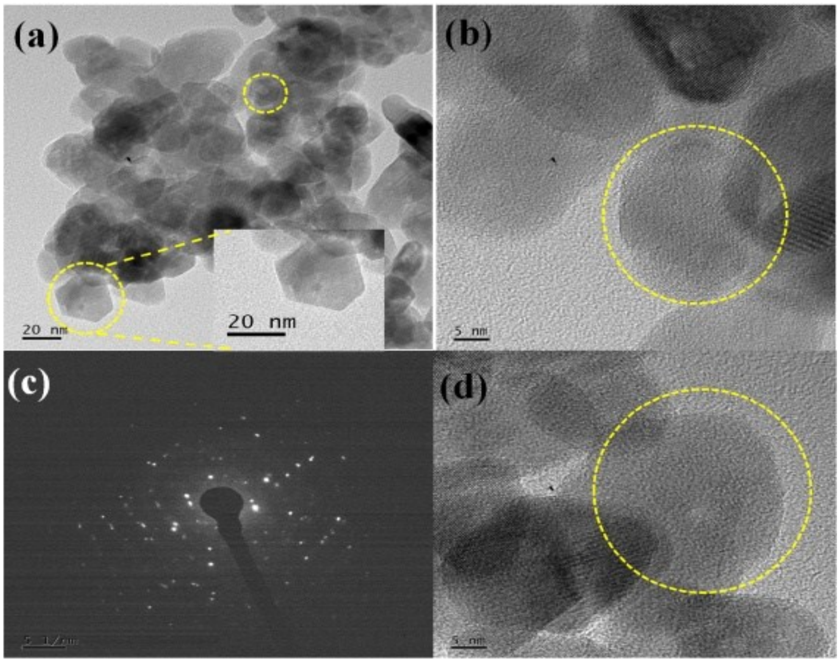

Figure 1. (a), (b) and (d) TEM image of 2D CuO nanoplates. (c) SAD pattern of CuO NPs having polycrystalline nature

Thermogravimetric Measurements:

Further the thermogravemetric analysis of as synthesized CuO catalysts is also carried out to understand the process of weight losses throughout calcination. The TGA depicts weight loss percentage with increase in temperature; **Fig.S1** is the thermogram of as synthesized samples. The thermogram depicts the weight loss (w_1) of 0.5 to 1 % below 150 °C corresponding to physically absorbed water [1]. The weight drop (w_2) of almost (1%) from 200 -500 °C corresponds to decomposition of Cu (II) to CuO. Further weight loss (w_3) up to the temperature of above 500 °C can be attributed to the thermal degradation of surfactant leaving 2.5-10 % of CuO.

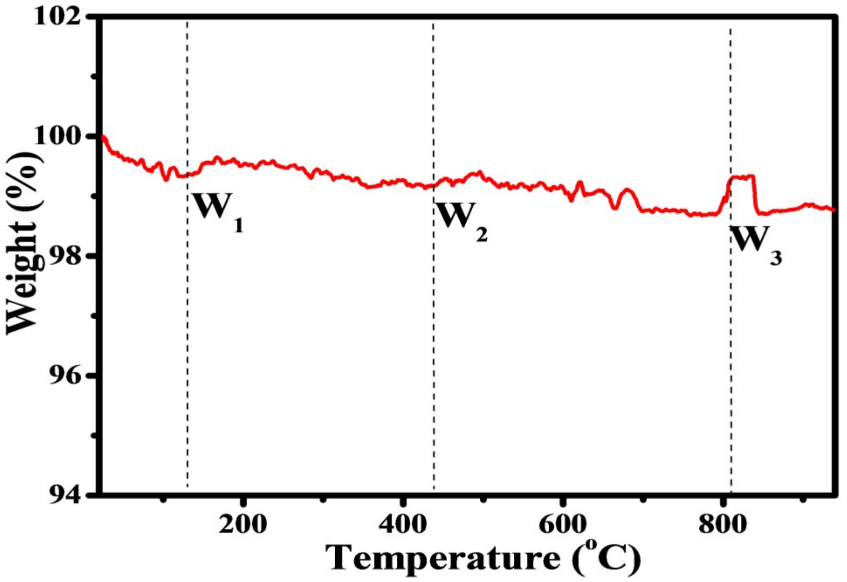

Fig.S1. TGA of the CuO NPs having three different losses corresponding to volatile solvent impurities along with moisture and surface bound PVP molecules respectively.

Nitrogen adsorption-desorption isotherm of CuO NPs recorded to evaluate the available surface area and the active sites available for chemical and electrochemical interaction of NO_2 and other active gases. **Fig. S2** indicates that the CuO NPs displays type II isotherm with

a hysteresis loop in the relative pressure (P/P_0) range from 0.8 to 0.9. The characteristic hysteresis loop indicates that the CuO is mesoporous in nature and the presence of mesopores could be due to the thermal decomposition of polyvinyl pyrrolidone (PVP) on CuO surface [2]. The BET (Brunauer-Emmett-Teller) surface area calculated from the adsorption branch of the isotherm is found to be $23.60m^2/gm$. The inset in **Fig. S2** illustrates the corresponding pore size distribution plot calculated by BJH (Barrett-Joyner-Halenda) from the adsorption data. The average pore radius and pore volume of the CuO NPS is calculated to be 2.3 nm and $2.599e^{-0.1}$cc/gm.

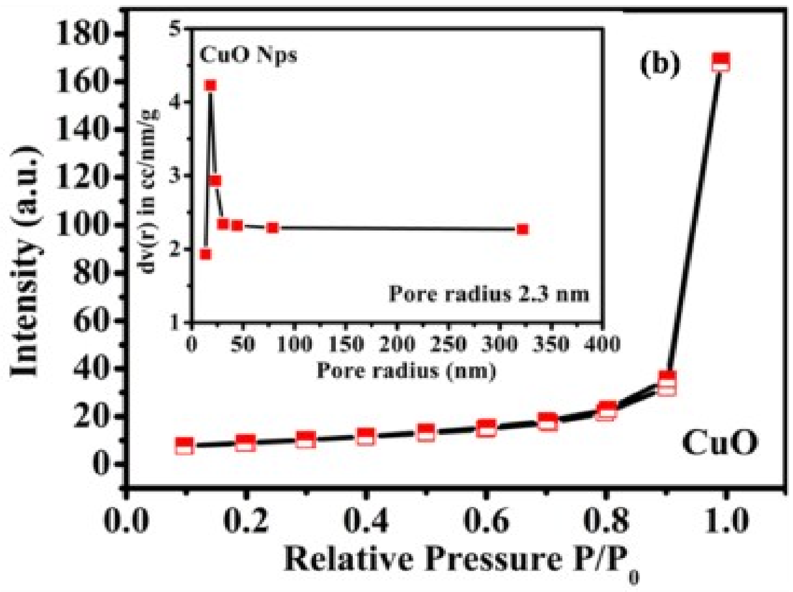

Fig. S2 . N_2 adsorption-desorption isotherm and BJH pore size distribution plot (inset) of annealed CuO thin films.

The response and recovery time is a significant concern for characterizing a sensor. The response and recovery times for CuO thin films to diverse concentrations of NO_2 were depicted from the electrical response curve **Fig S3 (a)**.

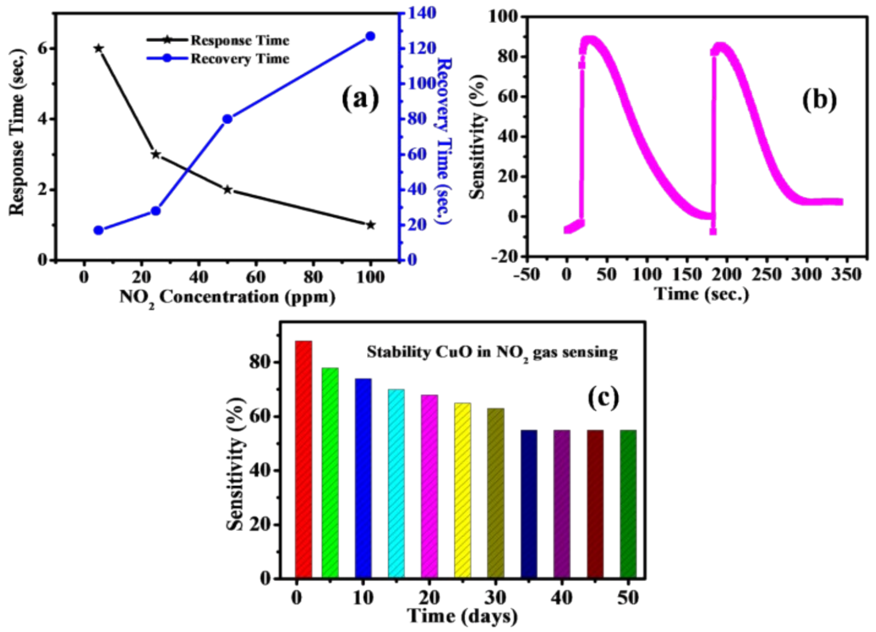

Fig. S3 (a) Variant of response and recovery time of the CuO thin film sensor with NO_2 concentration (b) Repeatability study of film to 100 ppm NO_2 gas, (c) Stability study of CuO thin film towards 100 ppm NO_2.

It was observed that as the concentration of NO_2 increases from 5-100 ppm, the response time decreases from 6 sec. to 1sec. and recovery times increases from 18 sec. to 130 sec. The decrease in response time could be due to the availability of vacant sites on thin film of the sensor for adsorption of gas, this may be probably due to, the reaction product's not leaving the oxygen surface immediately after the reaction and therefore the re-adsorption of oxygen is delayed, ensuing in a longer recovery time. NO_2 gas is a heavier gas it's recover longer times. This observed behaviour of increasing recovery time with increasing NO_2 concentration is similar to that of our previous reports on SnO_2 [3] and α-Fe2O3 [4]. Therefore, the repeatability of CuO NPs sensor has been studied further. The plot of repeatability study of CuO NPs sensor to fixed 100 ppm concentration of NO_2 gas is shown in **Fig. S3 (b)** exhibiting the excellent repeatability in response upon consecutive exposure of NO_2 gas.The stability in response of CuO NPs sensor at 150 °C is calculated for the period of 50 days and the corresponding results are presented in **Fig. S3 (c)**. Results indicate that the response rate of the CuO NPs sensor decreased from 88% to 64% (after the period of 35

days) with 72% stability. Further, it is noted that in case of metal oxide based gas sensors, the decrease in response rate with respect to time is predominantly due to aging effects [5].

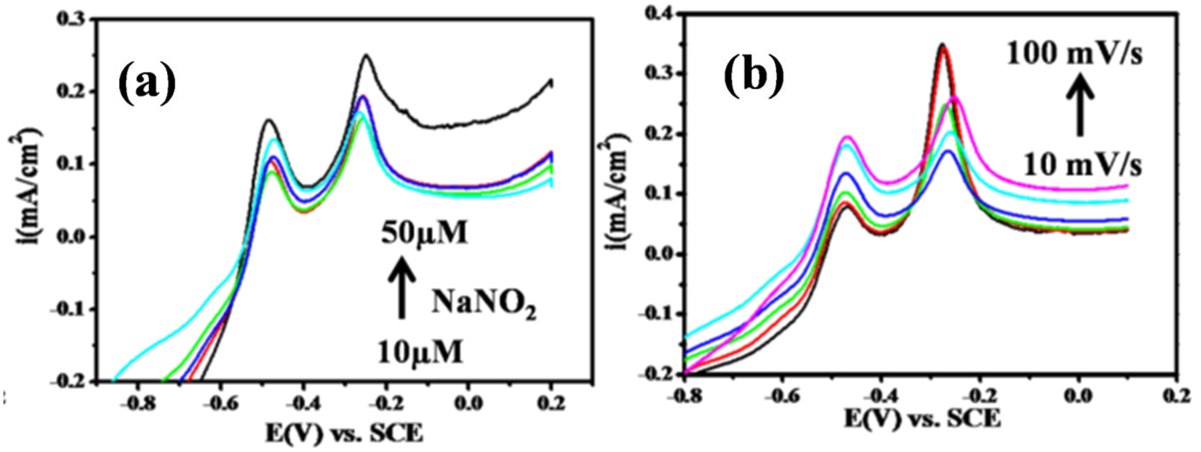

Fig. S4(a) LSV segment recorded for the CuO/GCE electrode at various concentrations of NO_2^- (10-50 μM) in 0.5M KOH at a scan rate of 50 mV/s. **(b)** CV of CuO/GCE in 0.5M KOH containing 30μM NO_2^- at different scan rates (10-100 mV/s).

The influence of the increase in concentration of NO_2^- on the electrocatalytic oxidation peak potential and peak current at the CuO NPs electrode in 0.5M KOH was studied using an LSV segment in **Fig. S4 (a).** The oxidation peak current at -0.30 V vs. RCE shows a linear response with the concentration of NO_2^- ions in the range of 10-50 μM and this linear range is broader than those of reported comparable electrocatalytic systems. Furthermore, the influence of the scan rate on the electrocatalytic oxidation peak potential (Epa) and peak current for 20 μM concentration NO_2^- at the CuO/GCE electrode in 0.5M KOH was studied using LSV, as shown in **Fig. S4 (b).** The current values were originate to increase with an increase in the scan rate from 10 to 100 mV/s **Fig. 4 (b).** The linear association between the anodic peak currents and shows that electrooxidation of NO_2^- is diffusion controlled.

References –

1. A. Kashale, K. A. Ghule, K. P. Gattu, V. H. Ingole, S. S. Dhanayat, R. Sharma, Y.-C. Ling, J. Y. Chang, M. M. Vadiyar, J Mater Sci: Mater Electron (2016)10854-016-5683
2. A. A. Kashale, K. P. Gattu, K. Ghule, V. H. Ingole, S. Dhanayat, R. Sharma, J. Y. Chang, A. V. Ghule, Composites Part B 99 (2016) 297-304
3. G. D. Khuspe, R. D. Sakhare, S. T. Navale, M. A. Chougule, R. N. Mulik, R. C. Pawar, C.S. Lee, V. B. Patil, Ceram. Inter. 39 (2013) 8673.
4. S.T. Navale, D.K. Bandgar, S.R. Nalage, G.D. Khuspe, M.A. Chougule, Y.D. Kolekar, S.Sen, V.B. Patil, Ceram. Inter. 39 (2013) 6453.
5. Y.H. Navale, S.T. Navale, N.S. Ramgir, F.J. Stadler, S.K. Gupta, D.K. Aswal, V.B. Patil, Sensors and Actuators B. 251 (2017) 551-563.
